# Author Correction: Regulating coordination number in atomically dispersed Pt species on defect-rich graphene for n-2 butane dehydrogenation reaction

**DOI:** 10.1038/s41467-021-24374-4

**Published:** 2021-06-30

**Authors:** Xiaowen Chen, Mi Peng, Xiangbin Cai, Yunlei Chen, Zhimin Jia, Yuchen Deng, Bingbao Mei, Zheng Jiang, Dequan Xiao, Xiaodong Wen, Ning Wang, Hongyang Liu, Ding Ma

**Affiliations:** 1 Shenyang National Laboratory for Materials Science, Institute of Metal Research, Chinese Academy of Sciences, Shenyang, P. R. China; 2 School of Materials Science and Engineering, University of Science and Technology of China, Shenyang, P. R. China; 3 Beijing National Laboratory for Molecular Sciences, College of Chemistry and Molecular Engineering and College of Engineering, and BIC-ESAT, Peking University, Beijing, P. R. China; 4 Department of Physics and Center for Quantum Materials, Hong Kong University of Science and Technology, Clear Water Bay, Kowloon, Hong Kong SAR, P. R. China; 5 State Key Laboratory of Coal Conversion, Institute Coal Chemistry, Chinese Academy of Sciences, Taiyuan, P. R. China; 6 University of Chinese Academy of Science, Beijing, P. R. China; 7 Shanghai Institute of Applied Physics, Chinese Academy of Sciences, Shanghai, P. R. China; 8 Center for Integrative Materials Discovery, Department of Chemistry and Chemical Engineering, University of New Haven, West Haven, CT USA

**Keywords:** Catalytic mechanisms, Heterogeneous catalysis, Chemical engineering

Correction to: *Nature Communications* 10.1038/s41467-021-22948-w, published online 11 May 2021.

The original version of this article inadvertently acknowledge Xiangbin Cai as a co-corresponding author instead of co-first author.

This has been corrected in both the PDF and HTML versions of the Article.

