# Supplementary material for: Regulating coordination number in atomically dispersed Pt species on defect-rich graphene for n-butane dehydrogenation reaction
Source: Nat Commun. 2021 May 11;12:2664. doi: 10.1038/s41467-021-22948-w (PMC8113322; doi:10.1038/s41467-021-22948-w)
Supplement: Supplementary file 1 — Supplementary Information [file 41467_2021_22948_MOESM1_ESM.pdf]

Supplementary Information

Regulating Coordination Number in Atomically Dispersed Pt Species on Defect-Rich Graphene for n-Butane Dehydrogenation Reaction

Xiaowen Chen[1, 2 #], *Mi Peng*[3 #], *Xiangbin Cai*[4 #], *Yunlei Chen*[5, 6 #], *Zhimin Jia*[1, 2], *Yuchen Deng*[3], *Bingbao Mei*[7], *Zheng Jiang*[7], *Dequan Xiao*[8], *Xiaodong Wen*[5, 6], *Ning Wang*[4*], *Hongyang Liu*[1, 2 *], *and Ding Ma*[3 *]

[1] Shenyang National Laboratory for Materials Science, Institute of Metal Research, Chinese Academy of Sciences, Shenyang 110016, P. R. China.

[2] School of Materials Science and Engineering, University of Science and Technology of China, Shenyang 110016, P. R. China.

[3] Beijing National Laboratory for Molecular Sciences, College of Chemistry and Molecular Engineering and College of Engineering, and BIC-ESAT, Peking University, Beijing 100871, P. R. China.

[4] Department of Physics and Center for Quantum Materials, Hong Kong University of Science and Technology, Clear Water Bay, Kowloon, Hong Kong SAR, P. R. China.

[5] State Key Laboratory of Coal Conversion, Institute Coal Chemistry, Chinese Academy of Sciences, Taiyuan 030001, P. R. China.

[6] University of Chinese Academy of Science, No. 19A Yuanquan Road, Beijing 100049, P. R. China.

[7] Shanghai Institute of Applied Physics, Chinese Academy of Sciences, Shanghai 201204, P. R. China.

[8] Center for Integrative Materials Discovery, Department of Chemistry and Chemical Engineering, University of New Haven, 300 Boston Post Road, West Haven, Connecticut 06516, United States.

[*] These authors contributed equally to this work.

Corresponding Author

*Email: liuhy@imr.ac.cn; phwang@ust.hk; dma@pku.edu.cn

Supplement Figures and Tables

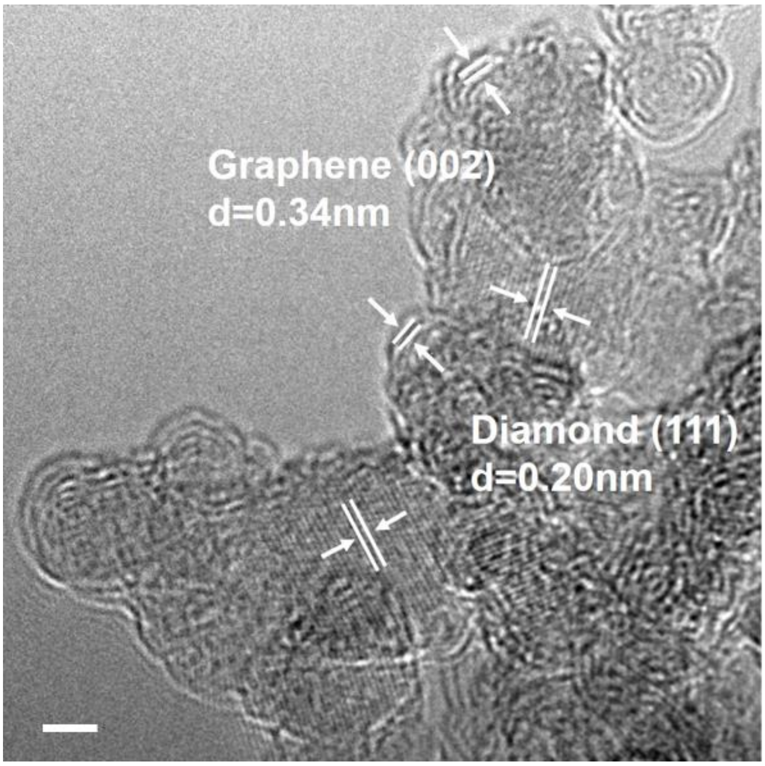

Supplementary Figure 1. HRTEM images ND@G, scale bar, 2 nm

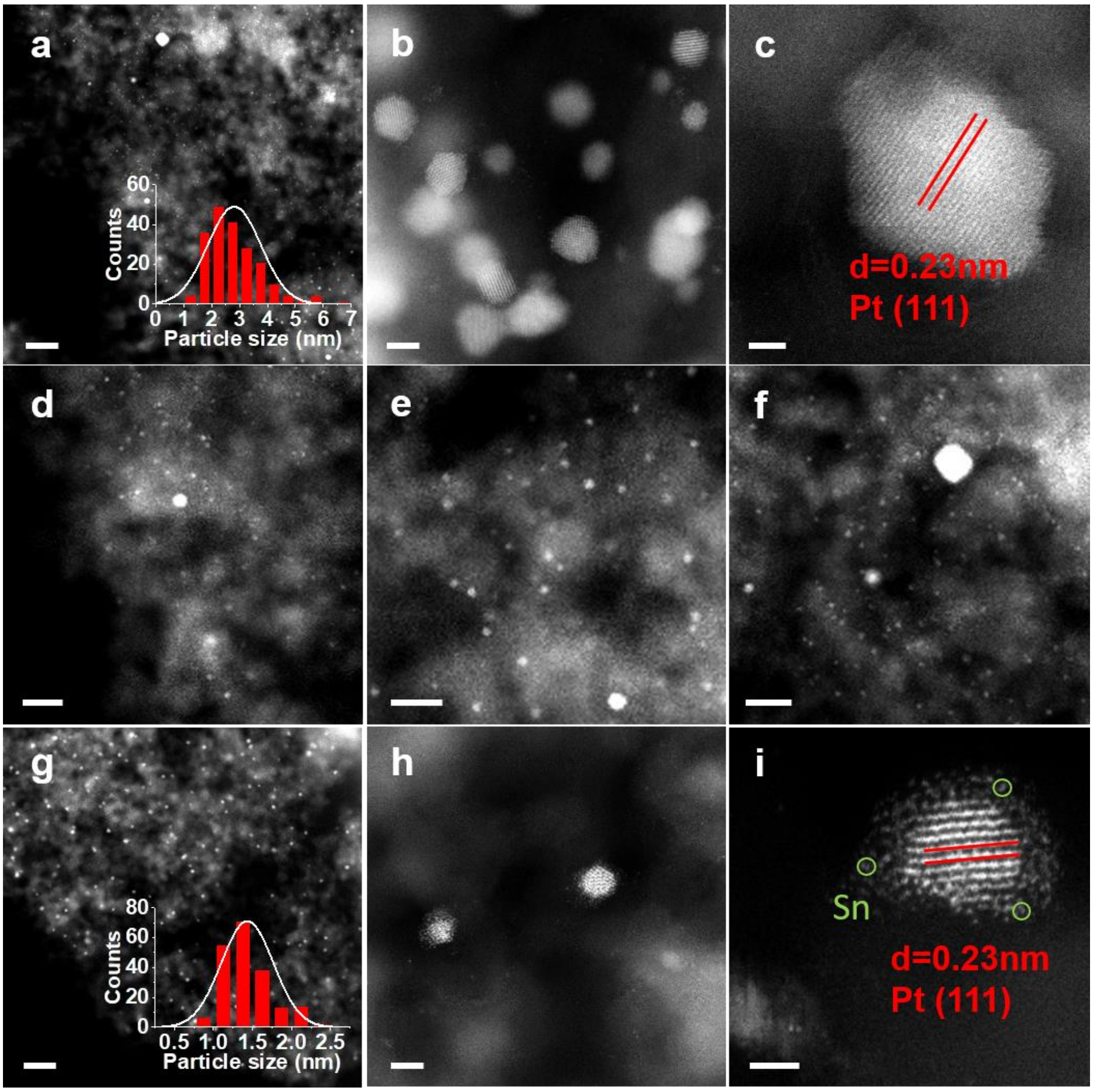

Supplementary Figure 2. Microscopic characterizations of Pt/ND@G and $Pt_{0.8Sn}$/ND@G. HAADF-STEM images of **(a)**-**(f)** Pt/ND@G and **(g)**-**(i)** $Pt_{0.8Sn}$/ND@G**.** The insets are particle size distribution of corresponding samples. In the images, atomically dispersed Sn atoms are highlighted by the green circles. Scale bars: **(a)**, **(d)**-**(g)** 10 nm; **(b)** and **(h)** 2nm; **(c)** and **(i)** 1 nm.

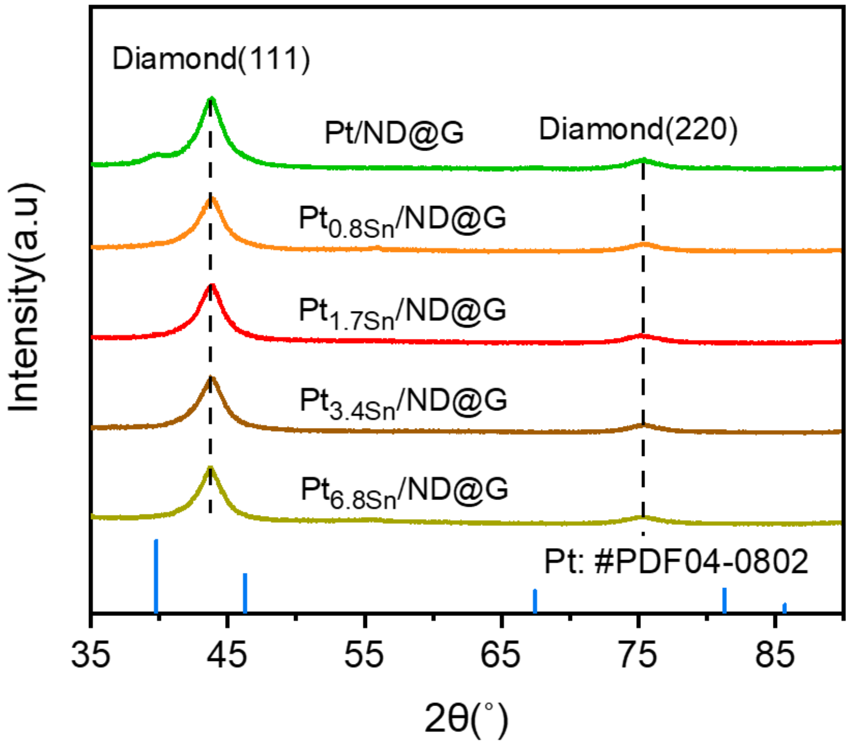

Supplementary Figure 3. XRD patterns of as-prepared catalysts.

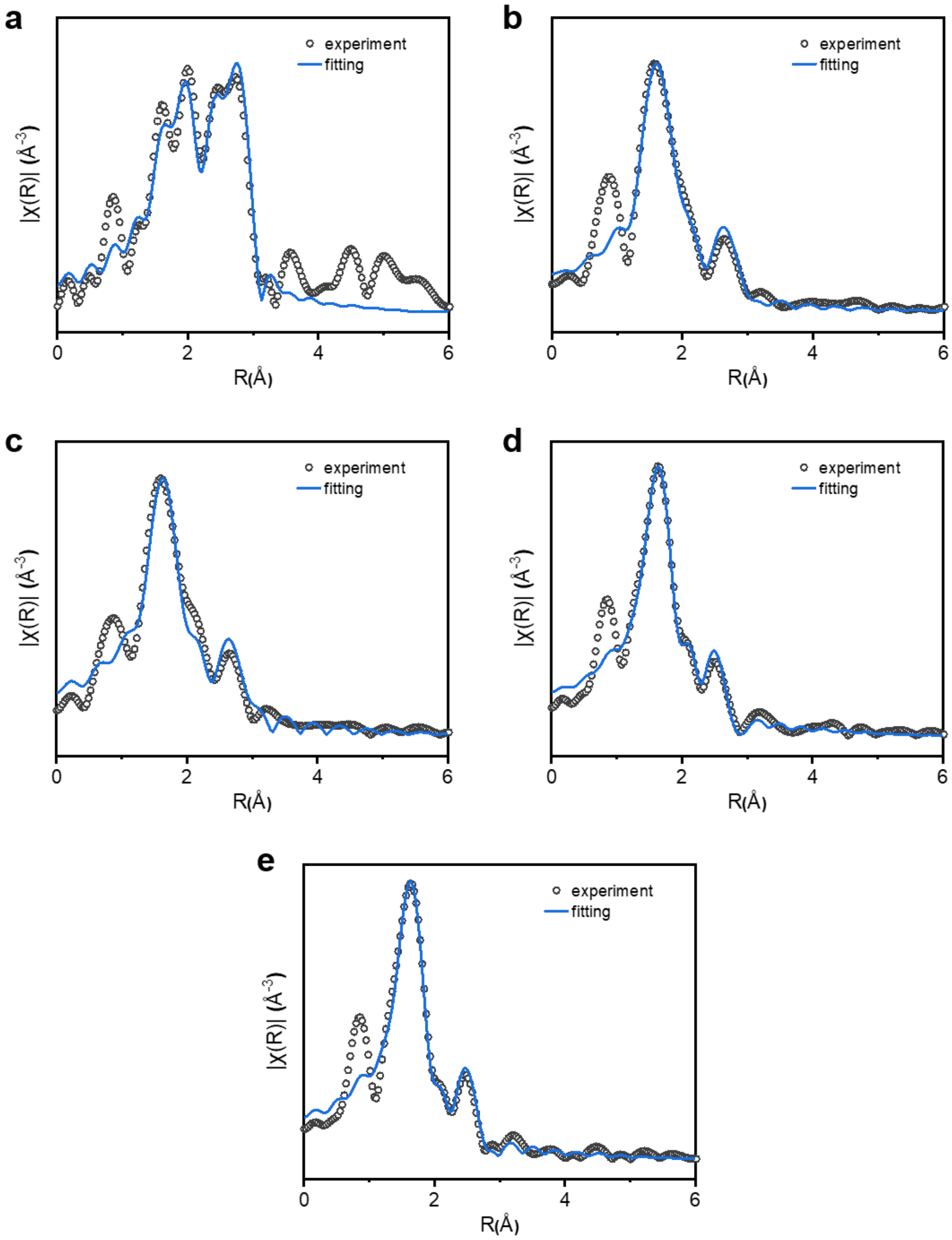

Supplementary Figure 4. Pt L_3-edge EXAFS fitting results for of as-prepared catalysts. **(a)** Pt/ND@G, **(b)** $Pt_{0.8Sn}$/ND@G, **(c)** $Pt_{1.7Sn}$/ND@G, **(d)** $Pt_{3.4Sn}$/ND@G and **(e)** $Pt_{6.8Sn}$/ND@G.

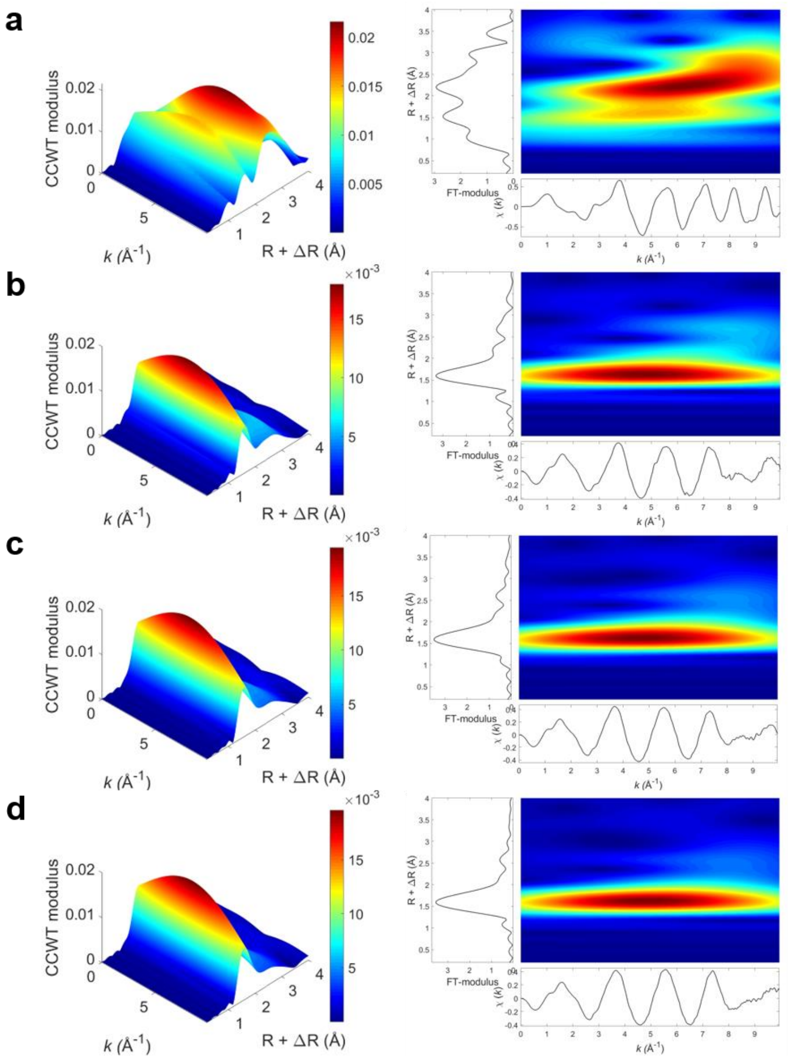

Supplementary Figure 5. Wavelet transform (WT) analysis of as-prepared catalysts. **(a)** Pt/ND@G, **(b)** $Pt_{0.8Sn}$/ND@G, **(c)** $Pt_{3.4Sn}$/ND@G and **(d)** $Pt_{6.8Sn}$/ND@G.

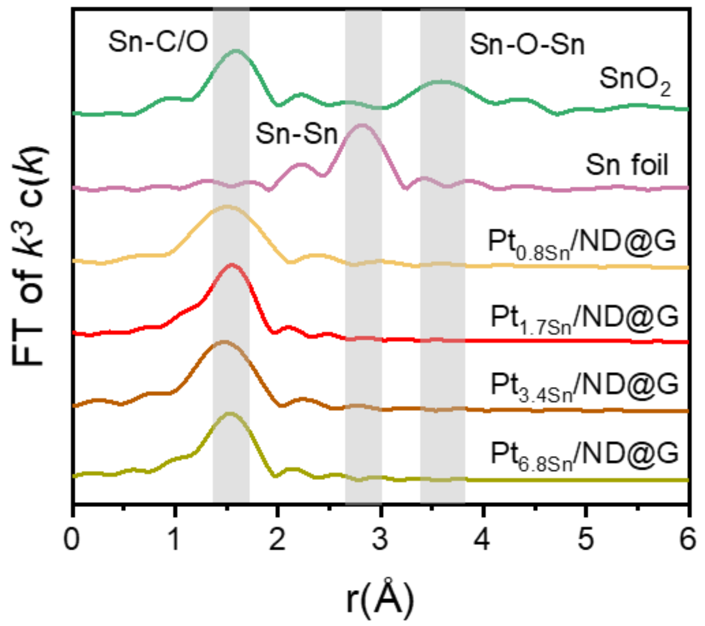

Supplementary Figure 6. FT-EXAFS profiles of Sn K-edge of as-prepared catalysts.

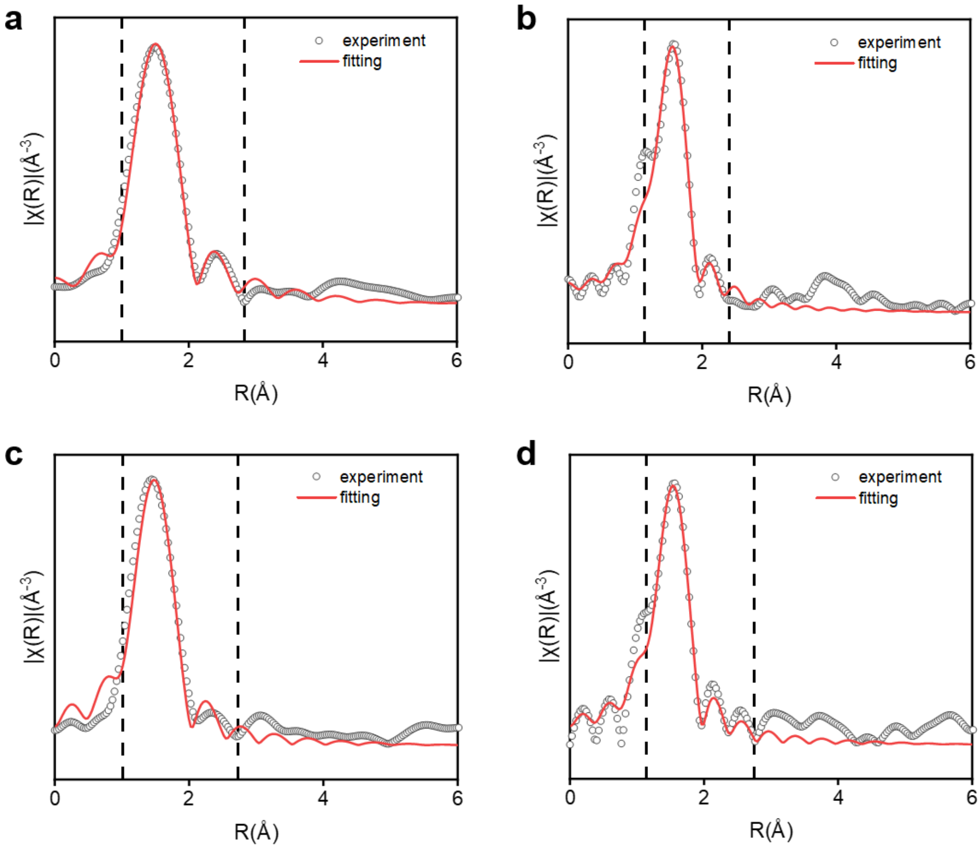

Supplementary Figure 7. EXAFS fitting results of Sn K-edge in R space. **(a)** $Pt_{0.8Sn}$/ND@G, **(b)** $Pt_{1.7Sn}$/ND@G, **(c)** $Pt_{3.4Sn}$/ND@G and **(d)** $Pt_{6.8Sn}$/ND@G.

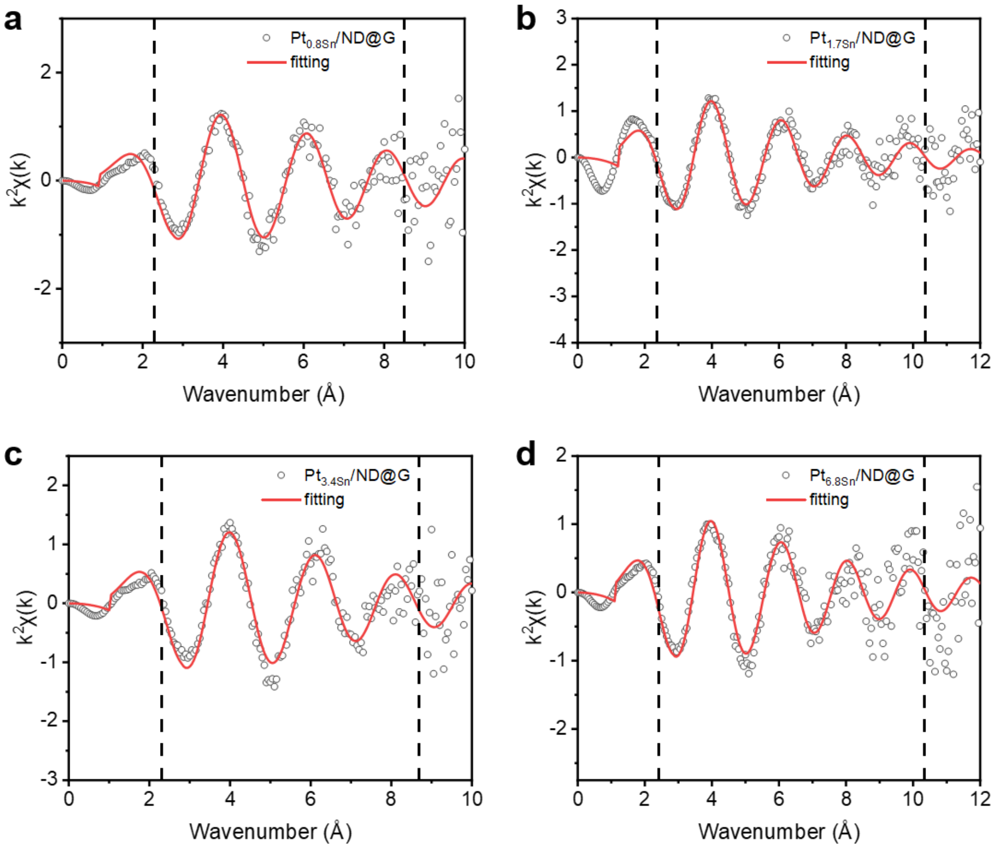

Supplementary Figure 8. EXAFS fitting results of Sn K-edge in k^2 space for as-prepared catalysts. **(a)** $Pt_{0.8Sn}$/ND@G, **(b)** $Pt_{1.7Sn}$/ND@G, **(c)** $Pt_{3.4Sn}$/ND@G and **(d)** $Pt_{6.8Sn}$/ND@G.

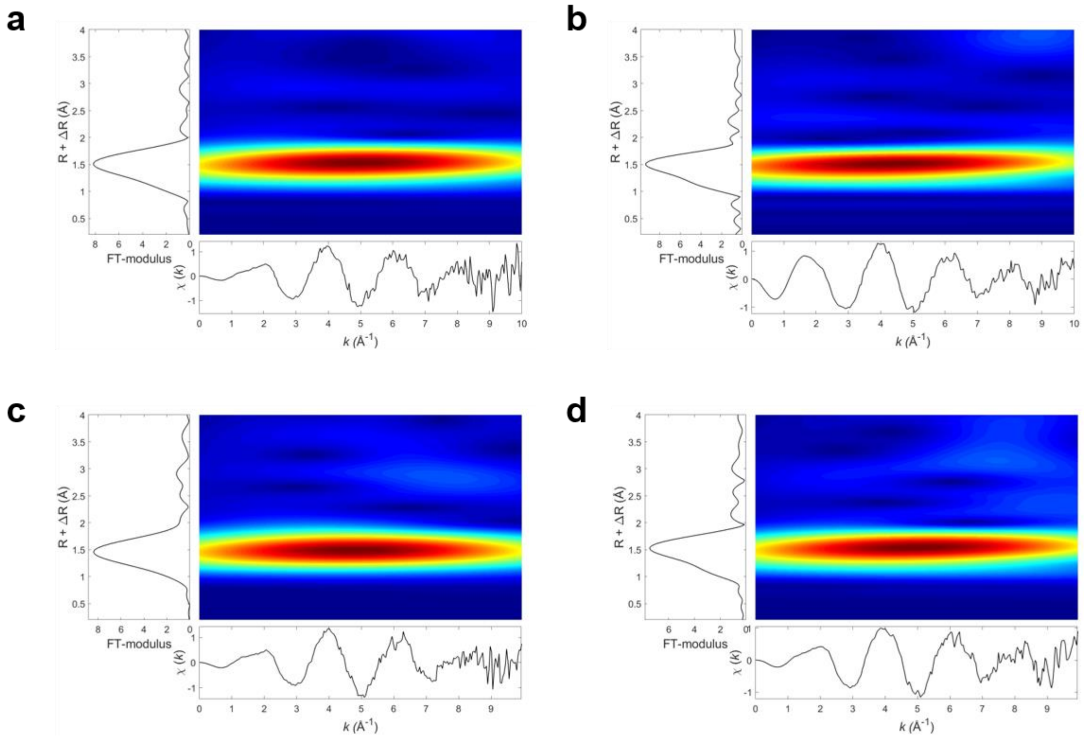

Supplementary Figure 9. WT analysis of as-prepared catalysts. **(a)** $Pt_{0.8Sn}$/ND@G, **(b)** $Pt_{1.7Sn}$/ND@G, **(c)** $Pt_{3.4Sn}$/ND@G and **(d)** $Pt_{6.8Sn}$/ND@G.

Supplementary Table 1. Sn K-edge EXAFS fitting results for as-prepared catalysts

Sample	Shell	C.N.	R(Å)	ΔE_0(eV)	S_0^2	R-factor
$Pt_{0.8Sn}$/ND@G	Sn-O/C	4.0	2.06	3.21	2.67	0.005
	Sn-Sn	-	-	-	-	-
	Sn-Pt	-	-	-	-	-
	Sn-O-Sn	-	-	-	-	-
$Pt_{1.7Sn}$/ND@G	Sn-O/C	4.2	2.07	5.51	4.27	0.01
	Sn-Sn	-	-	-	-	-
	Sn-Pt	-	-	-	-	-
	Sn-O-Sn	-	-	-	-	-
$Pt_{3.4Sn}$/ND@G	Sn-O/C	4.1	2.05	3.92	3.68	0.02
	Sn-Sn	-	-	-	-	-
	Sn-Pt	-	-	-	-	-
	Sn-O-Sn	-	-	-	-	-
$Pt_{6.8Sn}$/ND@G	Sn-O/C	3.4	2.07	2.89	4.99	0.02
	Sn-Sn	-	-	-	-	-
	Sn-Pt	-	-	-	-	-
	Sn-O-Sn	-	-	-	-	-

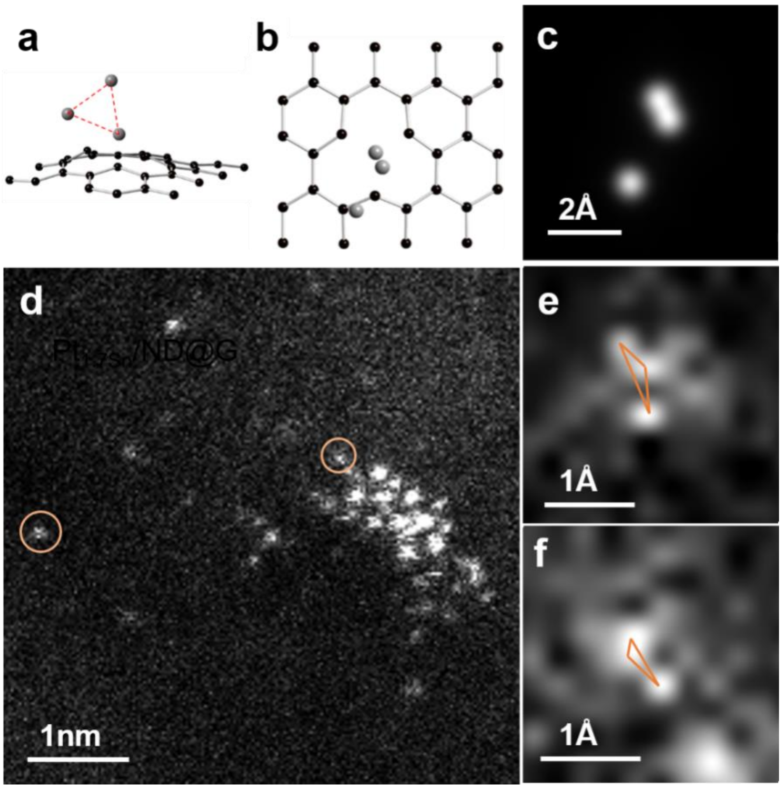

Supplementary Figure 10. Structural analysis of $Pt_{1.7Sn}$/ND@G. Perspective **(a)** and top **(b)** views of the DFT-computed Pt_3 cluster model, respectively. Black balls for carbon while gray balls for platinum. **(c)** Simulated STEM image according to the model. The graphene support is invisible under our experimental conditions. HAADF-STEM images **(d)** of $Pt_{1.7Sn}$/ND@G, the clusters highlighted by yellow circles enlarged in **(e)** and **(f).**

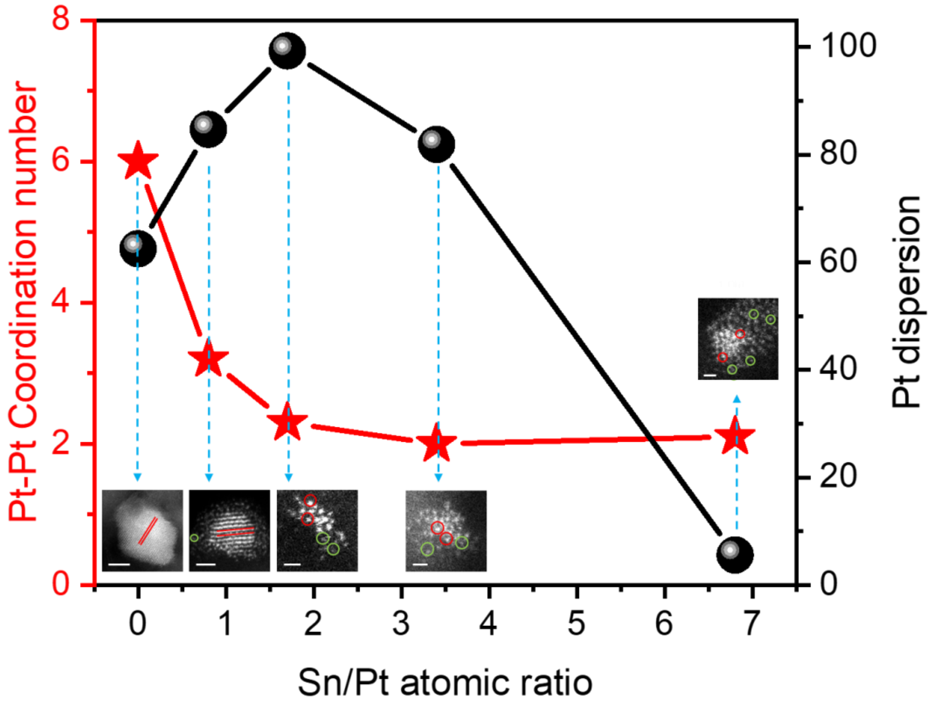

Supplementary Figure 11. Sn/Pt atomic ratio and Pt dispersion and Pt-Pt coordination number. The STEM images from left to right were Pt/ND@G, Pt0.8Sn/ND@G, Pt1.7Sn/ND@G, Pt3.4Sn/ND@G and Pt6.8Sn/ND@G, respectively. Scale bars: Pt/ND@G 2nm; Pt0.8Sn/ND@G 1nm; Pt1.7Sn/ND@G, Pt3.4Sn/ND@G and Pt6.8Sn/ND@G 5Å.

Supplementary Table 2. Activity properties of as-prepared catalysts for n-butane dehydrogenation reaction at 450 °C, GHSV=18000 $mL\cdot g\cdot cat^{-1}\cdot h^{-1}$

Catalyst	Conv. C_i/C_f (%)	Sele. S_i/S_f (%)[a]	Kd (h^{-1})[b]	τ (h)[c]	n-Butane rate ($mol\cdot g_{Pt}^{-1}\cdot h^{-1}$)
Pt/ND@G	11.6/8.2	96.0/97.8	0.0421	23.8	0.373
$Pt_{0.8}Sn$/ND@G	25.9/20.8	98.9/99.3	0.0313	31.9	0.833
$Pt_{1.7}Sn$/ND@G	35.4/30.9	99.0/99.3	0.0223	44.8	1.138
$Pt_{3.4}Sn$/ND@G	29.7/24.9	99.2/99.4	0.0265	37.7	0.951
$Pt_{6.8}Sn$/ND@G	2.4/0.8	100.0/100.0	0.1221	8.2	0.077

i: initial catalytic value after reaction 0.7h.

f: final catalytic value after reaction 9.83h.

[a]: Sele. C_4 olefin selectivity of the catalysts

[b]: k_d, deactivation rate constant, calculated from $kd=\{\ln[(1-C_f)/C_f]-\ln[(1-C_i)/C_i]\}/t$.

[c]: τ represents the catalyst life, which is the reciprocal of the deactivation rate constants ($\tau = 1/k_d$), and means time required for rates to decrease by e^{-1}.

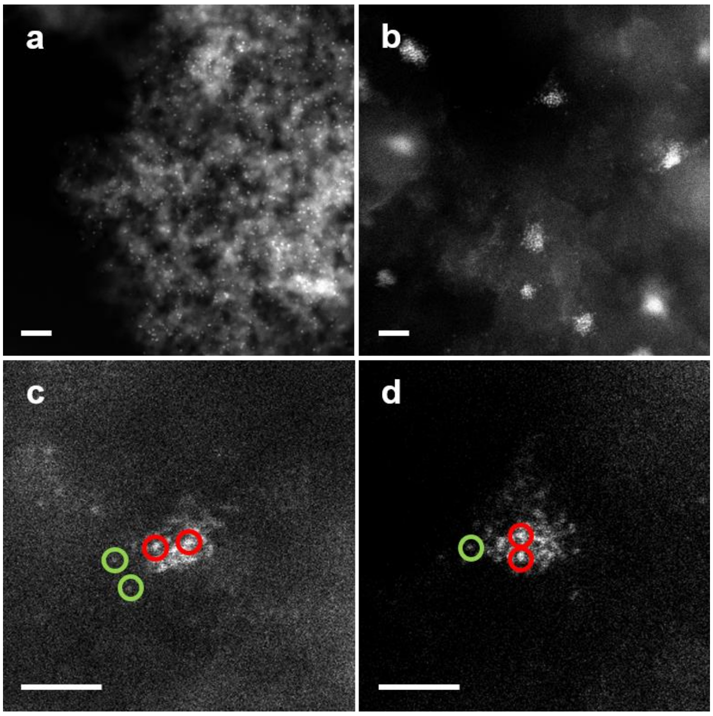

Supplementary Figure 12. HAADF-STEM images of the spent $Pt_{1.7Sn}$/ND@G. In the images, Pt clusters are highlighted by the red circles, and atomically dispersed Sn atoms are highlighted by the green circles. Scale bars: **(a)** 20nm; **(b)** 2 nm; **(c)**-**(d)** 1 nm.

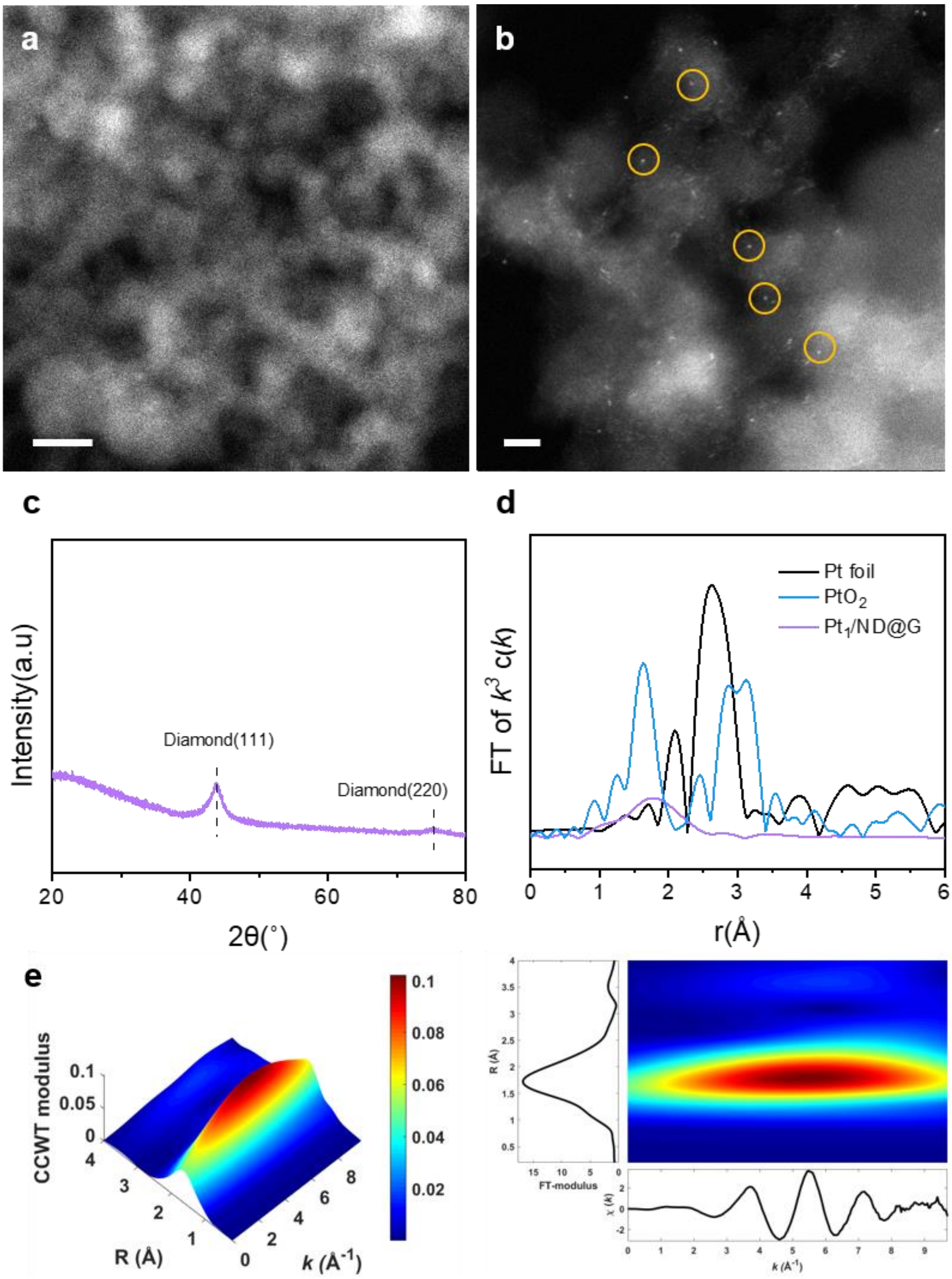

Supplementary Figure 13. Structural characterization of Pt_1/ND@G. **(a)** and **(b)** HAADF-STEM images of the Pt_1/ND@G. In the images, single Pt atoms are highlighted by the yellow circles. **(c)** XRD patterns of Pt_1/ND@G; **(d)** FT-EXAFS profiles of Pt_1/ND@G; **(e)** WT analysis of Pt_1/ND@G catalyst. Scale bars: **(a)** 10nm; **(b)** 2 nm.

Supplementary Table 3. Activity properties of as-prepared catalysts for n-butane dehydrogenation reaction at 450 °C, GHSV=18000 mL·g·cat^{-1}·h^{-1}

Catalyst	Conv. C_i (%)	n-Butene Sele. S_i (%)	n-Butane rate (mol·g_{Pt}^{-1}·h^{-1})	TOF(h^{-1})
Pt_n/ND@G	11.6	93.1	0.373	170.5
Pt_3/ND@G	35.4	96.6	1.138	423.2
Pt_1/ND@G	1.2	97.1	0.193	27.6

i: initial catalytic value after reaction 0.7h.

Computational Results of the Catalytic Mechanisms

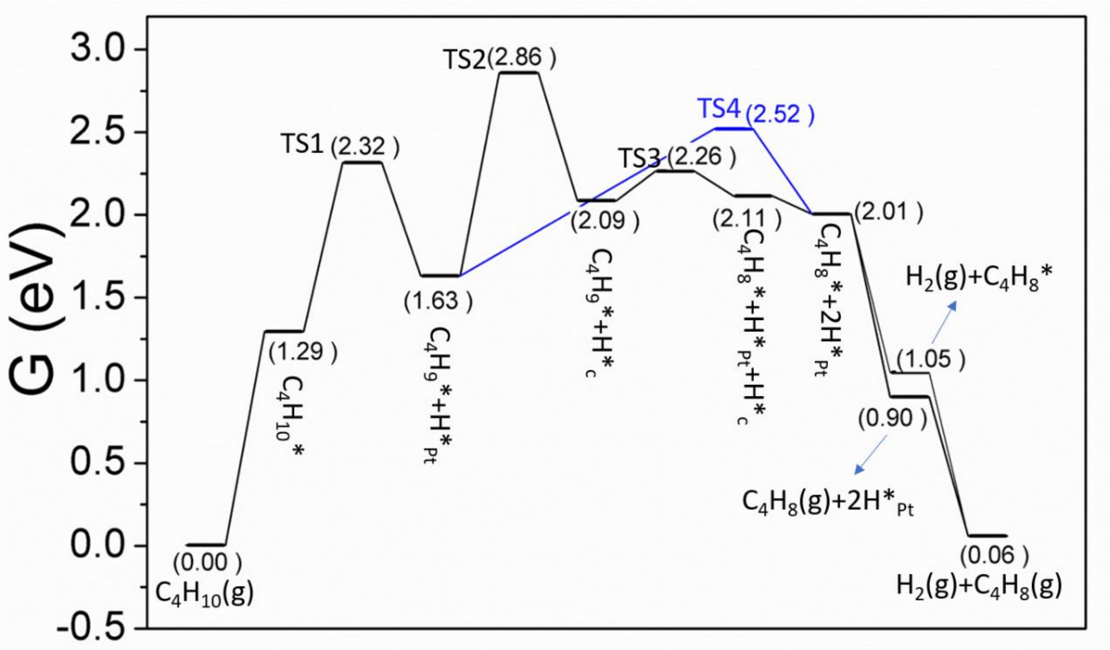

Supplementary Figure 14. Figure S1. Gibbs free energy profile of butane dehydrogenation to 2-butene on the Pt1-Gr.

Supplementary Table 4. Step by step barrier (E_a, eV) , reaction energy (ΔE, eV) , free energy barriers (Ga, eV) and reaction free energy (ΔG, eV) for n-butane dehydrogenation to 2-butene on Pt_1-Gr

Reactions		Ea/eV	ΔE/eV	Ga/eV	ΔG / eV
1	C_4H_{10}(g)->C_4H_{10}*	--	-0.05		1.29
2	C_4H_{10}*->2-C_4H_9*+H^*_{Pt}	0.96	0.34	0.96	0.34
3	2-C_4H_9*+H^*_{Pt}-> 2-C_4H_9*+H^*_C	1.22	0.45	1.22	0.45
4	2-C_4H_9*+H^*_C->2-C_4H_8*+H^*_{Pt}+H^*_C	0.18	0.03	0.18	0.03
5	2-C_4H_8(g)+H^*_{Pt}+H^*_C->2-C_4H_8(g) +2H^*_{Pt}	--	-0.11	--	-0.11
6	2-C_4H_8*+2H^*_{Pt}-> 2-C_4H_8* + H_2(g)	--	0.06	--	-0.96
7	2-C_4H_8*+2H^*_{Pt}->2-C_4H_8(g) + 2H^*_{Pt}	--	0.23	--	-1.11
8	2-C_4H_8* -> 2-C_4H_8(g)	--	0.35	--	-0.99
9	2H^*_{Pt} -> H_2(g)	--	0.18	--	-0.84
10	C_4H_8* ->2-C_4H_7*+ H*	0.80	0.76		

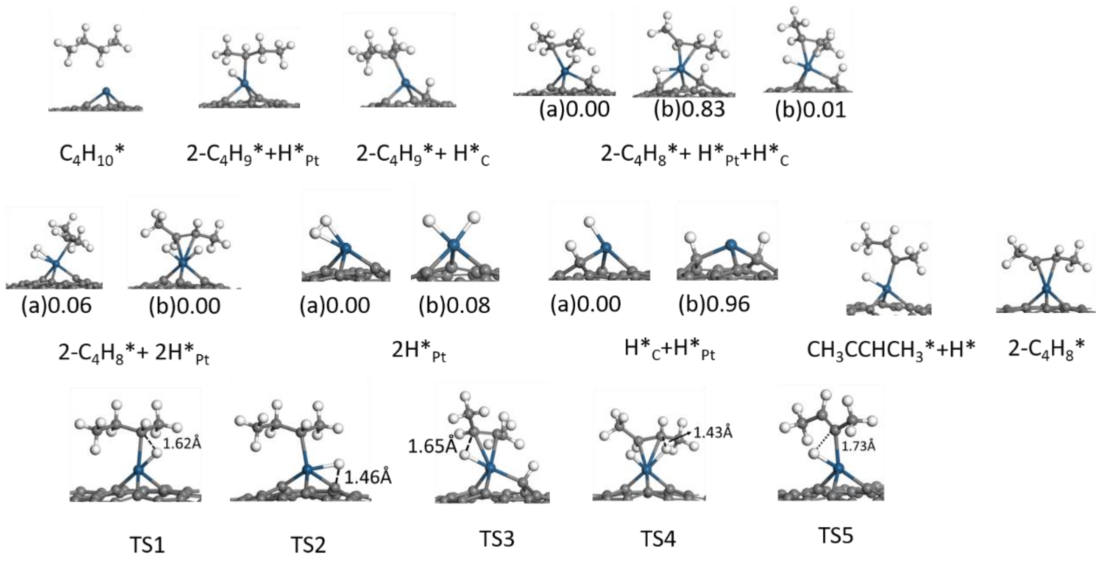

Supplementary Figure 15. All the possible configurations of surface intermediates on Pt_1-Gr catalyst. The relative energies of different configurations were listed below.

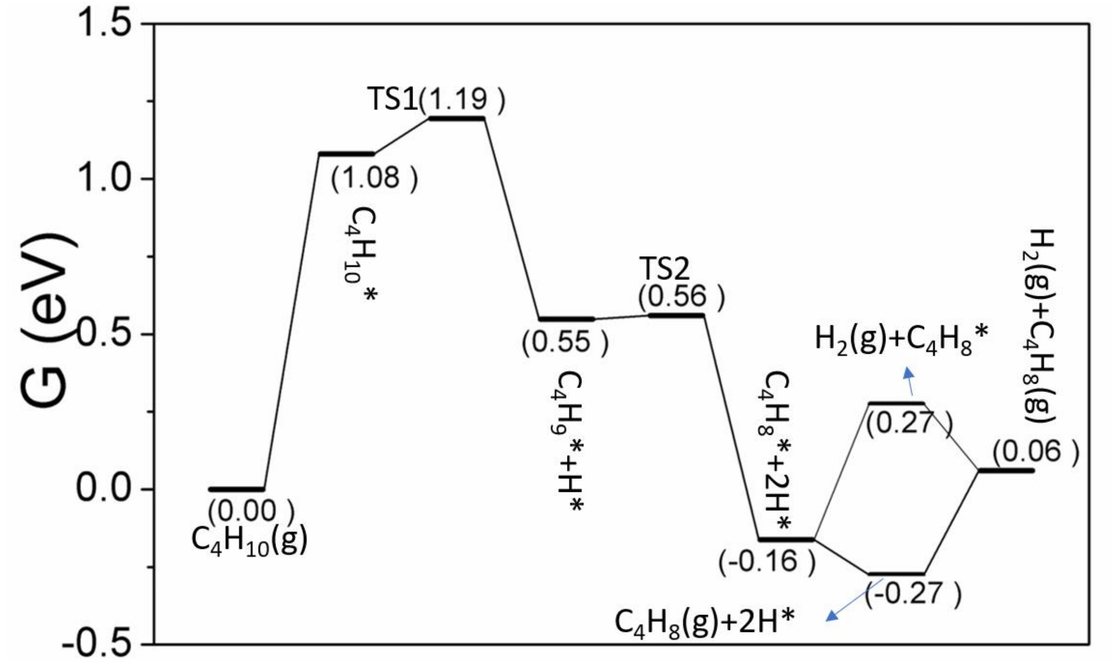

Supplementary Figure 16. Gibbs free energy profile of butane dehydrogenation to 2-butene on the Pt_3-Gr.

Supplementary Table 5. Step by step barrier (E_a, eV), reaction energy (ΔE, eV), free energy barriers (Ga, eV) and reaction free energy (ΔG, eV) for n-butane dehydrogenation to 2-butene on Pt_3-Gr.

Reactions	Ea / eV	ΔE / eV	Ga / eV	ΔG / eV
C_4H_{10}(gas) ->C_4H_{10}*	--	-0.26	--	1.08
C_4H_{10}* -> 2-C_4H_9*+H*	0.11	-0.53	0.11	-0.53
2-C_4H_9*+H*-> 2-C_4H_8*+2H*	0.01	-0.71	0.01	-0.71
2-C_4H_8*+2H*-> 2-C_4H_8(g)+2H*	--	1.23	--	-0.11
2-C_4H_8*+2H*-> 2-C_4H_8*+H_2(g)	--	1.46	--	0.44
2-C_4H_8*+2H*->CH_3CHCCH_3*+3H*	1.34	1.25	1.34	1.25
2H*-> H_2(g)	--	1.36	--	0.34

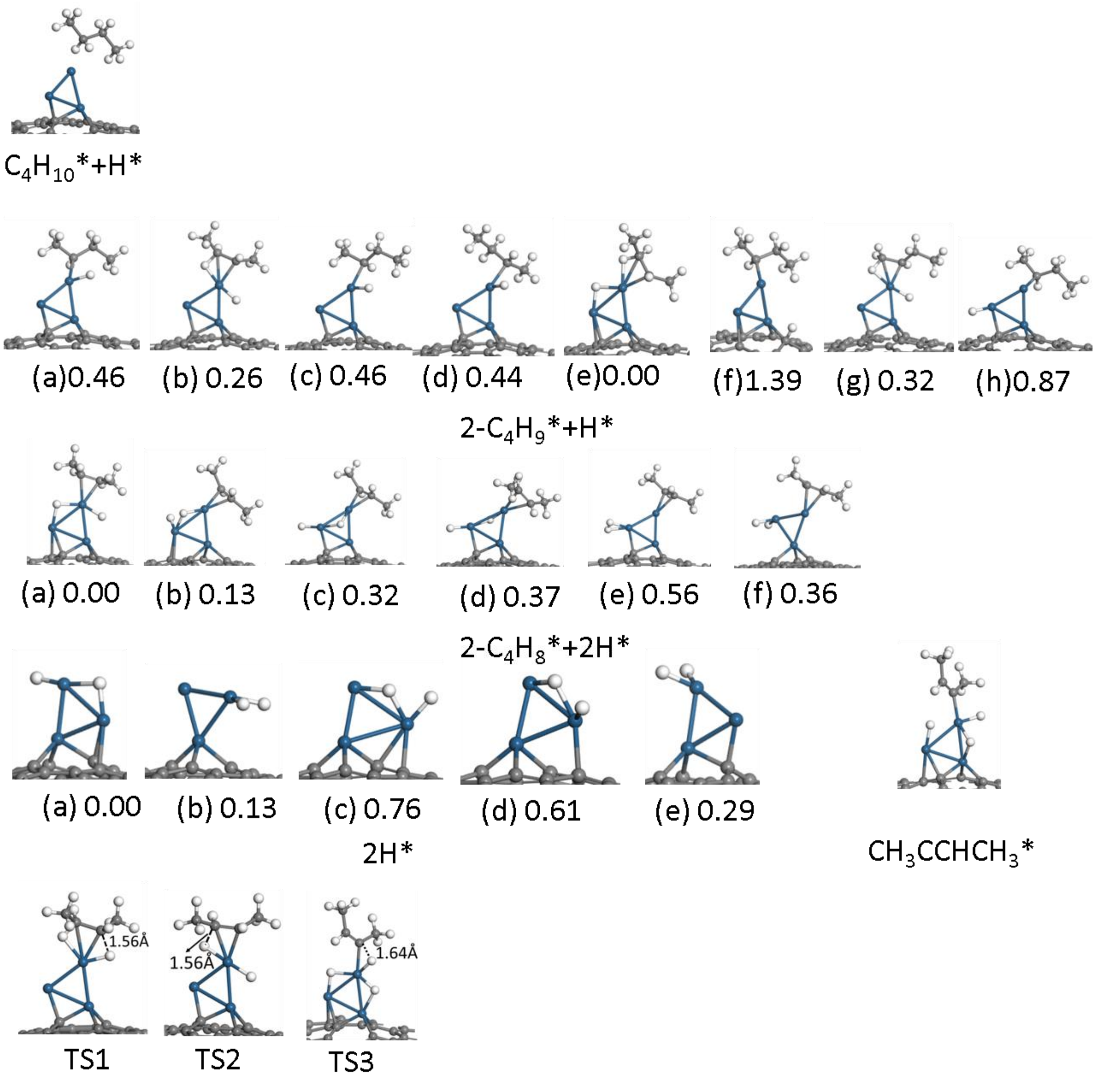

Supplementary Figure 17. All the possible configurations of surface intermediates on Pt_3-Gr catalyst. The relative energies of different configurations were listed below

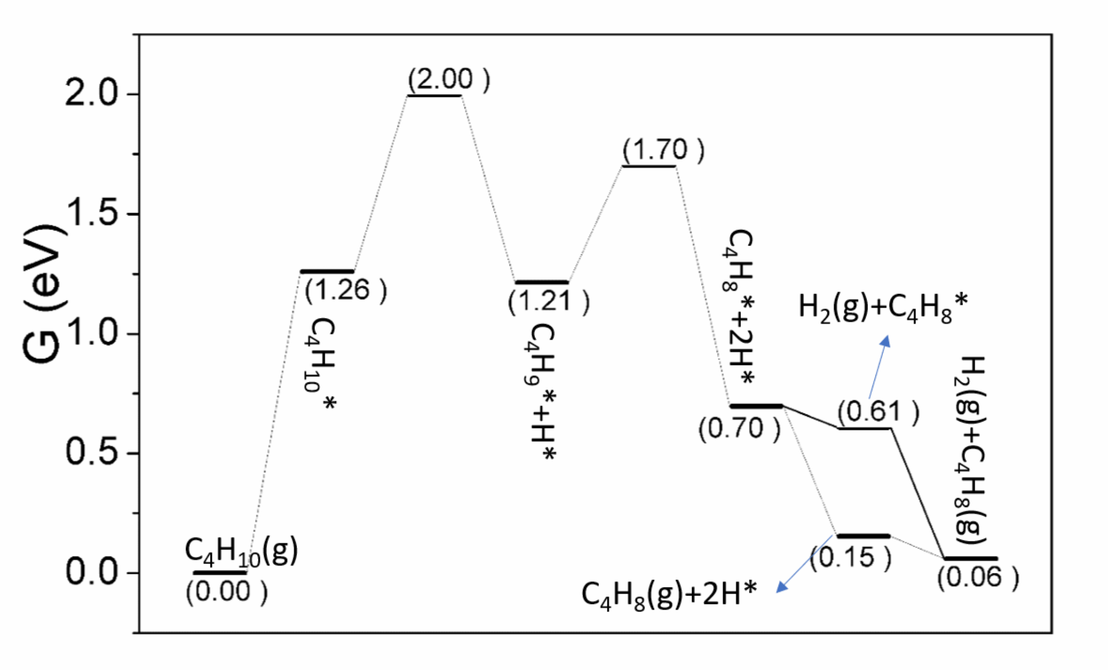

Supplementary Figure 18. Gibbs free energy profile of butane dehydrogenation to 2-butene on the Pt (111)

Supplementary Table 6. Step by step barrier (E_a, eV) and energy change (ΔE, eV) for n-butane dehydrogenation to 2-butene on Pt(111) surface

Reactions	Ea / eV	ΔE / eV	Ga / eV	ΔG / eV
C_4H_{10}(gas) -> C_4H_{10}*	--	-0.08		1.26
C_4H_{10}* -> 2-C_4H_9*+H*	0.74	-0.05	0.74	-0.05
2-C_4H_9* -> 2-C_4H_8*+H*	0.49	-0.51	0.49	-0.51
2-C_4H_8* -> 2-C_4H_8(gas)	--	0.79	--	-0.55
2-C_4H_8* -> CH_3CHCCH_3+H*	0.75	-0.01	0.75	-0.01
2H*-> H_2(g)	--	0.93	--	-0.09

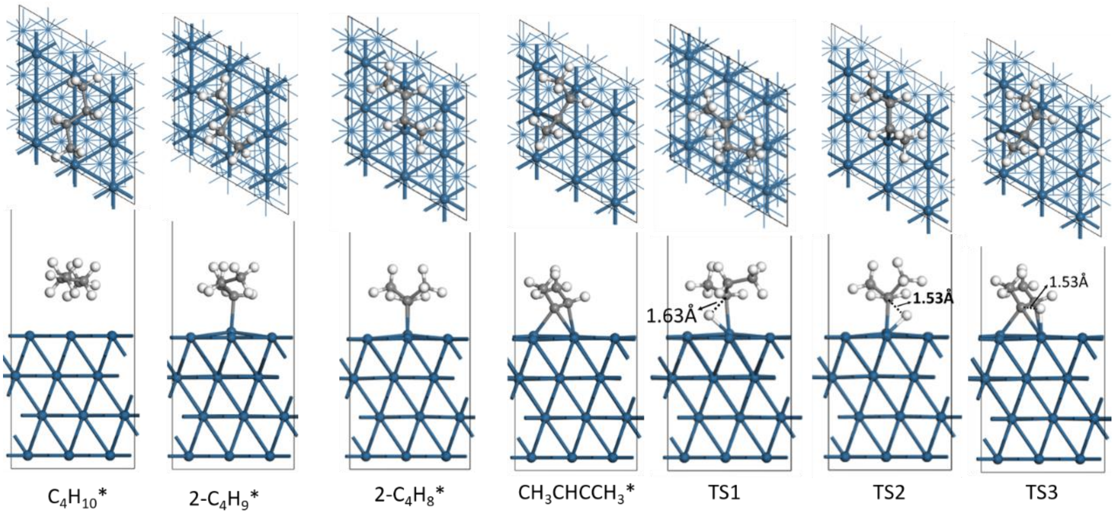

Supplementary Figure 19. All the configurations of surface intermediates on Pt(111) catalyst

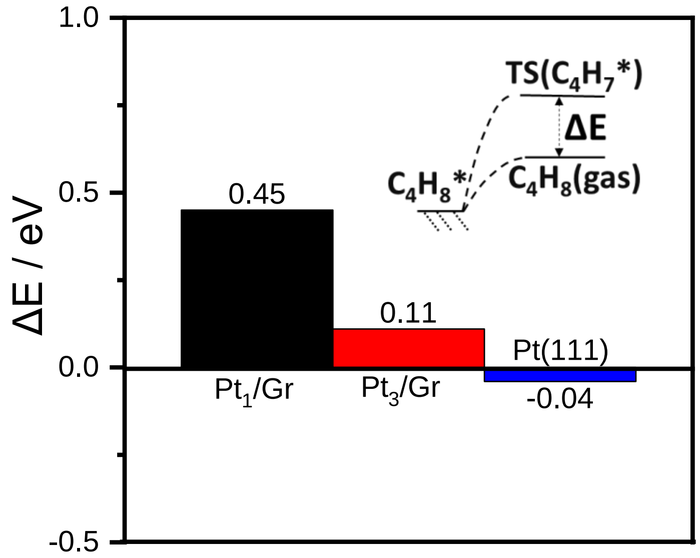

Supplementary Figure 20. The difference between the barrier of deep dehydrogenation (E_{DH}) and desorption (E_{DP}) of adsorbed 2-butene ($\Delta E_S = E_{DH} - E_{DP}$). It is used to evaluate the selectivity and stability of dehydrogenation from alkanes to alkenes, the more positive the value ΔE_S, the more stable and better selectivity of the catalyst.

It should be noted that, unlike the case of Pt_3-Gr and Pt(111), deep dehydrogenation is impossible for Pt_1-Gr because Pt_1-Gr can't further accommodate H atom after adsorbing two H atoms and 2-butene as shown in Figure S16, indicating the excellent stability and selectivity of dehydrogenation reaction, which verified our experimental observation.

Based on our calculated results, the chemical adsorption of H_2 molecule on Pt_1-Gr, Pt_3-Gr, and Pt(111) is 0.18, 1.36, 0.93 eV, respectively, indicating the adsorption H_2 on Pt1-Gr is quite weak and there may be no H coverage on Pt_1-Gr under reaction condition. Thus, we calculated ΔE_S for the only adsorbed 2-butene on Pt_1-Gr as shown in Figure S3(d) to evaluate its stability and selectivity. Interestingly, the calculated ΔE_S for only adsorbed 2-butene on Pt_1-Gr is 0.45 eV, much higher that of Pt_3-Gr and Pt(111), further verifying the good stability and selectivity of Pt_1-Gr catalysts.
